# Entropy Information of Pulse Dynamics in Three Stages of Pregnancy

**DOI:** 10.1155/2022/6542072

**Published:** 2022-10-14

**Authors:** Nan Li, Jiarui Yu, Luqi Huang, Xiaobo Mao, Yuping Zhao, Yi Yu

**Affiliations:** ^1^School of Electrical and Information Engineering, Zhengzhou University, Zhengzhou, China; ^2^China Academy of Chinese Medical Sciences, Beijing, China; ^3^The Obstetrics and Gynecology Hospital of Fudan University, Shanghai, China

## Abstract

The aim of the present study is to use entropy to explore the change of pulse generated by normal pregnant women with gestational. Firstly, the subjects were divided into early (E), middle (M), and late (L) three stages according to gestational age. Then, pulse signals of the Chi position of 90 pregnant women at different gestational ages were collected. Secondly, the four entropies, namely fuzzy entropy (FuEn), approximate entropy (ApEn), sample entropy (SamEn), and permutation entropy (PerEn), were applied to the analysis of the long-term pulse changes of the pregnancy. Finally, the related information about pulse in different stages of pregnancy is given by the analysis of four kinds of entropy. Furthermore, the statistical tests are conducted for further comparison, and the descriptive statistics and the results are presented. In addition, boxplots are employed to show the distribution of four entropies of pregnancy. This work has studied the changes in pulse during pregnancy from quantitative and qualitative aspects. Our results show that entropy improves the diagnostic value of pulse analysis during pregnancy and could be applied to facilitate noninvasive diagnosis of pregnant women's physiological signals in the future.

## 1. Introduction

Pulse feeling, reflecting the rate of the heartbeat, has its unique value in clinical application, which is confirmed by thousands of years of clinical application [1]. In general, there are four major diagnostic methods in traditional Chinese medicine (TCM): looking, listening, asking, and feeling the pulse. According to TCM methods, the rationale behind wrist-pulse-based diagnosis relies on the fact that blood flows through different organs at different rates, which may reflect different health statuses and can be identified according to the pulse fluctuation pattern [2].

**Figure 1:**
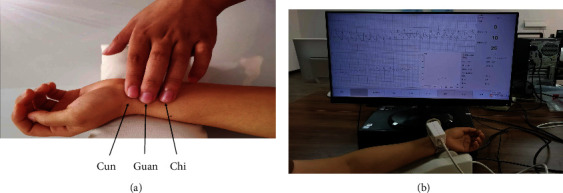
The pulse wave acquisition.

TCM divides the radial artery terminal area into three adjacent intervals, namely Cun, Guan, and Chi, whose positions are shown in Figure 1(a). The three positions of the radial artery correspond to the meridians and the visceral organs as illustrated in Table 1. The Cun positions of the left and right hands represent the heart and lungs, respectively. The Guan positions of the left and right hands reflect the health status of the liver and stomach, respectively. The Chi positions of both hands usually reflect the kidney condition. Therefore, the positions of Cun, Guan, and Chi have different meanings, which directly reflect the state of different organs of the human body.

Pulse condition is usually felt by TCM practitioners by placing their fingers on the three radial artery positions. The information acquired from radial artery is actually the comprehensive reflection of wave form, velocity, period, and swing of the pulse wave presented in radial artery [3, 4]. Therefore, a variety of physiological and pathological conditions, especially those of the cardiovascular system, can be detected by testing the pulse signal. At present, pulse diagnosis has been widely used in rehabilitation, disease diagnosis, constitution identification and clinical diagnosis, owing to its convenience and noninvasive nature [5, 6].

Pregnancy is a special state of that human body that is associated with substantial changes in the cardiovascular system of the mother. The pregnant woman undergoes significant physiological changes in order to nurture and accommodate the developing fetus during pregnancy. Due to physiological changes, the pulse condition will have a series of changes. These changes begin after conception and affect different organ systems in the body, including the cardiovascular, reproductive, circulatory, and urinary systems [7–10].

From the point of view of TCM, the kidney is closely related to the reproductive system, and the state of the kidney can be reflected by the pulse of the Chi position. Therefore, the pulse of the Chi position can better reflect the pulse during pregnancy. Furthermore, the performance of pregnancy pulse in the Chi position is particularly obvious as the gestation progresses. As stated in the classical work of TCM “Pulse Meridian”, the kidney is known as the cell door. The kidney stores the essence of the body and controls the reproduction system, while the Chi position can reflect the pulse of the kidney. Therefore, the diagnosis of pregnancy pulse should be mainly based on the Chi pulse and supplemented with the Cun and Guan pulse. This not only has a solid theoretical basis, but also has extensive clinical support. It is therefore important to understand the normal pulse changes during pregnancy, as this may help identify abnormal physiological changes. However, the pulse signals are extremely complex as they are influenced by many factors in the electrophysiology and the recording environment. The complexity of pulse signals makes it difficult to extract features and perform sequence analysis. Quantifying the dynamic irregularities of pulse series is still an important challenge in signal processing [11, 12].

Currently, many different methods concerning pulse analysis have been proposed by researchers, involving time domain analysis [13], frequency domain analysis [14], time-frequency joint analysis [15] and multifeature fusion analysis [16] etc. For example, Nie et al. used the mode energy ratio to analyse 83 female volunteers with different pregnancy statuses. They found that there is an incremental magnitude difference between the MER values of nonpregnancy, early pregnancy, and late pregnancy [17]. Su et al. adopted the Hilbert-Huang transform and instantaneous frequency to analyze radial artery pulse signals taken from women in their 36th week of pregnancy and after pregnancy [18]. Li et al. comprehensively investigated the changes in waveform characteristics of both photoplethysmographic (PPG) and radial pulses with gestational age during normal pregnancy. After normalizing the arterial pulse waveforms, the abscissa of notch point, the total pulse area, and the reflection index were extracted and compared between the PPG and radial pulses [7]. Liao et al. identify the difference among pulses and calculate the 10 relative energy values of the spectrum's harmonics of the positions in both wrists in three stages of pregnancy [19]. Kuo et al. obtained spectral indices of the pulse waveform and compared them among nonpregnant women and pregnant women in the three trimesters of pregnancy. The research found that the effects of pregnancy on the pulse wave are the reduction in the total power of the pulse and the power of higher order harmonics and the increase of lower order harmonics in the power spectrum of the pulse wave [20].

However, most of the studies focus on the time domain, frequency domain, and power analysis of the pulse during pregnancy, and few studies focus on the fluctuation state and regularity of the pulse in the three stages of pregnancy. Furthermore, these traditional methods based on linear concepts are restricted to system stability and have other deficiencies. For instance, the time domain analysis method is used to extract the characteristics of the pulse waveform, which is invalid for the small waveform changes caused by physiological changes. The frequency domain method can only describe the overall frequency characteristics of the signal and can not extract the local characteristics. They are not particularly sensitive to change of physiological state and are insufficient in characterizing the complex dynamics of nonlinear system. Therefore, methods of nonlinear analysis have been introduced to get a better insight into the complex signal.

In recent years, entropy has become a powerful tool to evaluate the regularity or complexity of time series because it provides estimation indexes without any assumptions about the underlying structure of the system [21–23]. The entropy methods derived from nonlinear dynamics have potential application to a wide variety of time series analyses [24]. The most popular entropy-based methods are approximate entropy (ApEn) and sample entropy (SamEn), which show potential applications in a wide range of physiological and clinical signals. Nie et al. used a piezoelectret pulse sensor to detect human pulses and the approximate entropy (ApEn) is utilized to analyse health conditions [25]. Liu et al. used the SamEn algorithm to acquire the chaotic features of the signals. After calculating the SamEn from the EEG signals, random forest was utilized for developing learning regression models with bispectral index (BIS) as the target [26].

Fuzzy entropy (FuEn), a new measure of time series regularity, was proposed and applied to the characterization of electromyography (EMG) signals. Vallejo et al. used discrete wavelet transform and FuEn to extract and select features from EMG signals, whereas artificial neural networks are used to give the recognition result [27]. Similar to the two existing related measures, ApEn and SamEn, FuEn is the negative natural logarithm of conditional probability, that is, for the next *m*+1 point, the vectors of two similar points remain similar. Generally, these three entropies are used to evaluate the repeatability of waveforms. The larger the entropy is, the more the frequencies in the waveform are disordered, and the smaller the entropy is, the smaller the disordered frequencies in the waveform are. Different from these three kinds of entropy, permutation entropy (PerEn) mainly considers the sudden change of waveform or information. It is a method to detect the dynamics of mutation and the randomness of time series and can quantitatively evaluate the random noise contained in the signal sequence [28]. Furthermore, PerEn estimates the relative frequency of ordered patterns extracted from time series and introduces the concept of Shannon entropy (ShaEn) into the analysis of ordered patterns [29]. Clearly, the entropy analysis superiors to most nonlinear dynamic measures in some degree, such as fractal dimension [30], Lyapunov spectrum [31], Kolmogorov-Sinai (KS) entropy [32] etc. To the best of the author's knowledge, the research on the gestation period problem is still open and remains challenging.

Motivated by the above idea, we employ the entropy to disclose some pulse clinical value of the gestation period. There are several points in our study that significantly differ from the existing studies. We have studied the correlation and difference of pulse in different pregnancy, as well as the change law of pregnancy pulse as a proof of TCM concept. Firstly, we used a related measure of time series regularity, entropy, and applied it to characterizing pregnancy pulse signals. The variability and fluctuation trend of the long-term pregnancy pulse waveform in different stages were calculated. Secondly, the pulse changes are explained from the perspective of repeatability and mutation, etc. of pregnancy pulse by using four different entropies. Furthermore, the significance analysis of pulse in three different gestational periods is given, which proves the correctness of the TCM theory about the difference of pulse in different pregnancy periods. Finally, this study has quantified the pulse waveform characteristic differences in terms of pulse entropy among the three trimesters.

The rest of the paper is organized as follows. In Section 2, some preliminary work and methodology are introduced. In Section 3, the experimental results are explicitly presented. In Section 4, the analysis and discussion are given, respectively. Finally, some future work is given in Section 5.

## 2. Methodology

### 2.1. Data Collection

In this study, pulse waveform recordings were available from 90 volunteers at the Affiliated Obstetrics and Gynecology Hospital of Fudan University, who were divided into three trimesters of pregnancy (8–12 weeks; 24–28 weeks; and 32–34 weeks). The basic physiological data of the participants are shown in Table 2. Each of the participants understood the purpose of the study. The research received the ethics approval from the Affiliated Obstetrics and Gynecology Hospital of Fudan University. All pregnant women met the following inclusion criteria: (1) a normal menstrual cycle; (2) no chronic hypertension, diabetes, or cardiovascular disease; (3) normal liver and kidney function; and (4) not taking blood pressure drugs.

The wrist pulse measurements were performed in a quiet clinical measurement room at the Affiliated Obstetrics and Gynecology Hospital of Fudan University, Shanghai, China. All pregnant women were asked to sit quietly for 5 minutes to achieve a stable heart rate so as to ensure the authenticity and effectiveness of the collected data. The pulse sampling frequency is 220 Hz. A pressure sensor was placed on the Chi position of the left wrist to record the radial pulse for 5 minutes using a DSOI-C data collection system. The waveform acquisition is shown in Figure 1.

### 2.2. Data Preprocessing

**Figure 2:**
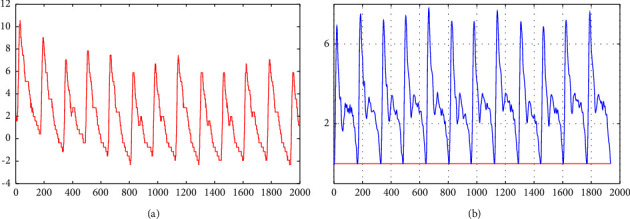
Pulse wave preprocessed.

The pulse signal will inevitably be polluted by the subjects' breathing, artifact movement, and other factors in the process of pulse acquisition. Therefore, preprocessing is the key to reducing noise and eliminating baseline drift of the pulse waveform before further analysis. In this paper, a robust signal preprocessing framework and a cascade adaptive filter based on wavelet were used to denoise and remove baseline drift, respectively. The original pulse wave and pulse wave preprocessed are shown in Figure 2.

### 2.3. Fuzzy Entropy (FuEn)

Fuzzy entropy (FuEn) describes the degree of fuzziness of a fuzzy set. The larger the fuzzy entropy is, the greater the sequence complexity is. For an *M* sample time series {*u*(*i*), 1 ≤ *i* ≤ *M*}, given *n*, form vector sequences {*X*_*i*_^*m*^, *i*=1,…, *M* − *n*}, as follows:

(1) $$\begin{array}{c} X_{j}^{m}=\left\{u\left(j\right),u\left(j+1\text{,}\right),\ldots ,u\left(j+n-1\right)\right\}-u_{0}\left(j\right), \end{array}$$

where {*u*(*i*), *u*(*j*+1),…, *u*(*i*)} represents *n* consecutive *u* values from *jth* point,

(2) $$\begin{array}{c} u_{0}\left(j\right)=\frac{1}{n}\overset{n-1}{\underset{j=0}{\sum }}u\left(i+j\right). \end{array}$$

For two *n*-D vector, *X*_*j*_^*n*^ and *X*_*j*_^*n*^, define the distance *d*_*ij*_^*n*^ between *X*_*j*_^*n*^ and *X*_*j*_^*n*^ as the maximum absolute difference of the corresponding scalar components

(3) $$\begin{array}{c} d_{ij}^{n}=d\left[X_{i}^{n},X_{j}^{n}\right]=\max\left|u\left(j+k\right)-u_{0}\left(j\right)-u\left(i+k\right)-u_{0}\left(i\right)\right|. \end{array}$$

The similarity degree *D*_*ij*_^*n*^ of *X*_*i*_^*n*^ and *X*_*j*_^*n*^ was defined using fuzzy function *μ*(*d*_*ij*_^*n*^, *m*, *r*)

(4) $$\begin{array}{c} D_{ij}^{n}=\mu \left(d_{ij}^{n},m,r\right)=\exp\;\left(-{\left(d_{ij}^{n}\right)}^{m}/r\right). \end{array}$$

Define the function *ϕ*^*n*^(*m*, *r*) as

(5) $$\begin{array}{c} \phi^{n}\left(m,r\right)=\frac{1}{M-n}\overset{M-n}{\underset{i=0}{\sum }}\left(\frac{1}{M-n-1}\overset{M-n}{\underset{j=1,j\neq i}{\sum }}D_{ij}^{n}\right). \end{array}$$

Similarly, get the function *ϕ*^*n*+1^(*m*, *r*)

(6) $$\begin{array}{c} \phi^{n+1}\left(m,r\right)=\frac{1}{M-n}\overset{M-n}{\underset{i=0}{\sum }}\left(\frac{1}{M-n-1}\overset{M-n}{\underset{j=1,j\neq i}{\sum }}D_{ij}^{n+1}\right). \end{array}$$

Finally, the FuEn can be defined as follows:

(7) $$\begin{array}{c} FuEn\left(m,n,r\right)=\lim_{M⟶\infty }\;\left[\ln\;\phi^{n}\left(m,r\right)-\ln\;\phi^{n+1}\left(m,r\right)\right], \end{array}$$

Which, for finite datasets, can be estimated by the statistics

(8) $$\begin{array}{c} FuEn\left(m,n,r,N\right)=\left[\ln\;\phi^{n}\left(m,r\right)-\ln\;\phi^{n+1}\left(m,r\right)\right]. \end{array}$$

### 2.4. Approximate Entropy (ApEn)

Approximate entropy is a measure that quantifies complexity and organization, which is a family of statistics that provides a measure of regularity, closely related to the Kolmogorov entropy. For example, a low ApEn means a high degree of regularity. ApEn has been repeatedly recommended as a relative measure for comparing data sets. The obvious advantage of this method is its capability to discern changing complexity from a relatively small amount of data and better outlier rejection ability. When this statistic is used to compare time series for similar epochs, more frequent and more similar epochs lead to lower values of ApEn [33]. Therefore, we consider ApEn to be particularly suitable for revealing pulse changes in pregnancy progression. For a description of ApEn, we refer to a time series, *x*(*i*), *i*=1,…, *N*.

(9) $$\begin{array}{c} y\left(i\right)=\left\{x\left(i\right),x\left(i+1\right),\ldots ,x\left(i+m-1\right)\text{,}\right\} \end{array}$$

The Euclidean distance *d*{*y*(*i*), *y*(*j*)} between any component of *y*(*i*) and *y*(*j*) is calculated, and the maximum distance between each component is defined as the maximum contribution component distance *D*{*y*(*i*), *y*(*j*)}.

(10) $$\begin{array}{c} D\left\{y\left(i\right),y\left(j\right)\right\}=\max\;\left\{y\left(i+k\right)-y\left(j+k\right)\right\} \end{array}$$

Given tolerance *r*, let *B*_*i*_ be the number of vectors *x*_*m*_(*j*) with in *r* of *x*_*m*_(*i*). The empirical probability *C*_*i*_^*m*^(*r*) that a vector *x*_*m*_(*j*) is within *r* of *x*_*m*_(*i*) can be estimated by

(11) $$\begin{array}{c} C_{i}^{m}\left(r\right)=\frac{B_{i}}{N-m+1}. \end{array}$$

After introducing Φ_*m*_^*N*^(*r*), given as

(12) $$\begin{array}{c} \Phi_{m}^{N}\left(r\right)=\frac{\overset{N-m+1}{\underset{i=1}{\sum }}\ln\left[C_{i}^{m}\left(r\right)\right]}{N-m+1}. \end{array}$$

ApEn can be defined as

(13) $$\begin{array}{c} ApEn\left(m,r\right)=\lim_{N⟶\infty }\;\left[\Phi_{m}^{N}\left(r\right)-\Phi_{m+1}^{N}\left(r\right)\right], \end{array}$$

which, for a finite time series, is estimated as

(14) $$\begin{array}{c} ApEn\left(m,r,N\right)=\Phi_{m}^{N}\left(r\right)-\Phi_{m+1}^{N}\left(r\right). \end{array}$$

ApEn(*m*, *r*, *N*) is computed according to three parameters, *m*, *r*, and *N*. Where, *m* is the dimension of the signal that will be expanded; *r* is the threshold; and *N* is the signals length to be computed. Both theoretical analysis and clinical practices concluded that *m* = 1 or 2, and *r* is between 10% and 25% of the standard derivation of the data to be analyzed, produces good statistical validity of ApEn(*m*, *r*, *N*).

### 2.5. Sample Entropy (SamEn)

Sample entropy measures the complexity of time series by measuring the probability of generating new patterns in signals. The greater the probability of generating new patterns is, the greater the complexity of time series is. Assume we have a real-valued discrete time series of length *N*: *x*={*x*_1_, *x*_1_,…, *x*_*N*_}. A set of *m*-dimensional vector sequences are composed by serial number, which is

(15) $$\begin{array}{c} Xm\left(i\right)=\left\{x\left(i\right),x\left(i!\right),\ldots ,x\left(i+m-1\right)\right\}. \end{array}$$

Define the distance between such vectors as the maximum difference of their corresponding scalar components,

(16) $$\begin{array}{c} d\left[X_{m}\left(i\right),X_{m}\left(j\right)\right]=max_{k=0,\ldots ,m-1}\left|x\left(i+k\right)-x\left(j+k\right)\right|. \end{array}$$

A match happens when the distance *d*[*X*_*m*_(*i*), *X*_*m*_(*j*)] is smaller than a predefined tolerance *r*. The probability *B*^*m*^(*r*) shows the total number of *m*-dimensional matched vectors. Similarly, *B*^*m*+1^(*r*) is defined for embedding dimension of *m*+1. Finally, the SamEn is defined as follows:

(17) $$\begin{array}{c} SamEn\left(m,r\right)=\lim_{M⟶\infty }\;\left\{-\ln\left[\frac{A_{m}\left(r\right)}{B_{m}\left(r\right)}\right]\right\}. \end{array}$$

When *n* is a finite value, the sample entropy is

(18) $$\begin{array}{c} SamEn\left(m,r,N\right)=-\ln\left[\frac{A_{m}\left(r\right)}{B_{m}\left(r\right)}\right]. \end{array}$$

### 2.6. Permutation Entropy (PerEn)

The permutation entropy algorithm is a dynamic mutation detection method, which can easily and accurately locate the mutation time of the system and can amplify the small changes in the signal. By reconstructing the phase space of a group of time series *X* with length *N*, the matrix *Y* is obtained. Each reconstructed component is rearranged in ascending order, and the column index of each element position in the vector is obtained to form a set of symbol sequences. Calculate the number of occurrences of each sequence of symbols divided by m! The total number of occurrences of different symbol sequences is taken as the probability of the reconstruction component. The calculation formula of permutation entropy of time series *x* is as follows:

(19) $$\begin{array}{c} PerEn=-\overset{k}{\underset{j=1}{\sum }}P_{j}\ln\left[P_{j}\right]. \end{array}$$

The maximum value of permutation entropy is ln (*d*!). The permutation entropy is normalized as follows:

(20) $$\begin{array}{c} 0\leq PerEn=\frac{PerEn}{\ln\;\left(d!\right)}\leq 1. \end{array}$$

The size of permutation entropy indicates the randomness of time series *x*, the smaller the entropy value is, the simpler and more regular the time series is. On the contrary, the larger the entropy, the more complex and random the time series.

## 3. Experiments

**Figure 3:**
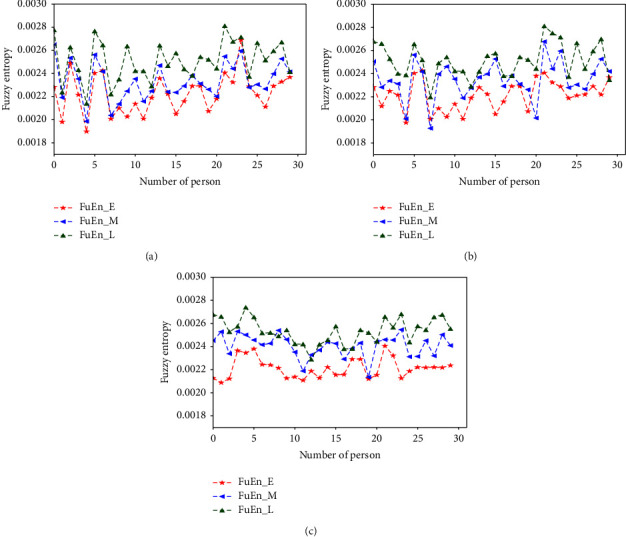
FuEn values of different pregnancy periods.

In this section, a series of comparative experiments were conducted. All subjects completed the experimental trials and measurements. The four statistics of FuEn, ApEn, SamEn, and PerEn were firstly tested on uniform random numbers. In order to better illustrate the relationship between entropy and sampling points, 3000, 5000, and 8000 pulse data were used to calculate entropyvalues and analyzed,

respectively Figure 3–Figure 10 show the performance of FuEn (*x*,1,0.25,2), ApEn (*x*,1,0.15), SamEn (*x*,2,0.2,1), and PerEn (*x*,3,1,1) on random 30 numbers, respectively. The performances of the four statistics of FuEn, ApEn, SamEn, and PerEn were analyzed and the conclusions were given, respectively. The four entropy values of the *E*, M, and L stages of pregnancy are given in Table 3.

### 3.1. FuEn Analyses and Comparisons

Having applied the FuEn analysis of three stages of pregnancy pulses, the FuEn average values of these three stages do have a significant difference. Figure 3 illustrates the values of FuEn in different pregnancy periods. Each stage contains 30 subjects, and their FuEn means all vary from 0.002 to 0.028. Clearly, the mean values of the E stage are smaller than the M stage and the L stage. As shown in Figure 3, the red polyline represents the FuEn value of the E stage of pregnancy, which ranges from 0.002 to 0.0025. The blue line represents the FuEn value in the second trimester of pregnancy, in which the mean value is about 0.0023, slightly higher than that in the first trimester of pregnancy. In the third trimester of pregnancy, the values of FuEn are the largest, which is about 0.0024 to 0.0028. The longer the pregnancy time, the greater the values of FuEn. From Figure 3, you can find that the FuEn of the L stage fluctuates bigger and more similarly, which means that the pulse in this stage fluctuates more irregularly.

### 3.2. ApEn Analyses and Comparisons

Based on the ApEn, Figure 5 shows the characteristics of the pulse movement during pregnancy. The graph distinguishes the ApEn values of the three stages of pregnancy, in which the values of entropy in the three stages are very different. Usually, the mean value of ApEn in E stage is smaller than the M stage's, and the value of he M stage is less than that of the L stage. Notably, most of the ApEn values in the E stage of pregnancy were concentrated in the range of [0.7–0.8]. The ApEn of the M stage is slightly higher than that of the E stage of pregnancy, as is shown in Figure 5 by the blue curve and the red curve, respectively. The maximum ApEn appears in the L stage of pregnancy, where the average value is about 0.9. All of the approximate entropies are typical enough to stand for their groups, respectively. The analysis indicates that the longer the gestational weeks, the more variable the pulse will be.

### 3.3. SamEn Analyses and Comparisons

The subject curve profiles of SamEn for E, M, and L stages are shown in Figure 7 by the red line, blue line, and green line, respectively. There is an obvious tendency to increase in the M stage of pregnancy compared with the E and L stages. From this figure, it is noted that the pulse series of the M stage shows a larger number of cases exhibiting nonlinear dynamics in comparison with those of the E and L stages. The sample entropy values in the E and L stages of pregnancy are mostly concentrated in the region of [0.007–0.008], while the value of the M stage jumps to about 0.009. Additionally, it is important to note that the percentage of pulse series found for both the E stage and the L stage is quite similar.

### 3.4. PerEn Analyses and Comparisons

PerEn was used to analyze the variability of pulse in three stages of pregnancy, and a significant difference among the three stages can be found. Compared with the three entropies mentioned above, the PerEn has the opposite trend. As seen in Figure 9, we find that a higher value of entropy is assigned to the pulse series from the E stage, and the values of entropy decrease in the M and L stages, which is consistent with the hypothesis of mutagenicity with gestational period. The E stage's PerEn mean value is higher than the M and L stages'. The PerEn variabilities of these three stages do have significant differences. Clearly, you can find that most of the PerEn of the E stage are in the region of [0.75–0.85], as shown in Figure 9. However, the entropy values of the M stage and the L stage are between [0.65–0.75] and [0.65–0.70], respectively. The comparison between Figures 9 and 10 shows the consistency of the PerEn. Therefore, not only the specific values of the entropy measure need to be analysis but also their tendency in different stages need to be taken into account to better characterize a physiologic process.

## 4. Analysis and Discussion

### 4.1. Statistical Analysis

In order to judge the difference among the three physiological states of the E, M and L stages, the statistical analysis was performed for statistical differences among three stages. One-way analysis of variance (ANOVA) was used to analyze the difference degree with a significance *p* = 0.05. The multiple comparison of quantitative data is processed by the analysis of variance (LSD test).

Statistical tests were conducted for further comparison, and descriptive statistics and the results are presented in Table 3. In the table, data are expressed as mean ± standard deviation. As can be seen, the results obtained with these four entropy measures are in good correspondence. Through the analysis of significant *p* value, it can be concluded that *p* value is less than 0.05, which indicates that there is a significance difference in the three stages of pregnancy. Obvious, it provides a theoretical basis for quantitative and qualitative analysis of the pulse characteristics of pregnancy.

### 4.2. Influence of the Amount of Data on Entropy

In order to better explain the influence of the amount of data on entropy, entropy values of 3000, 5000, and 8000 sampled pulse points were calculated and analyzed, respectively. Figures 3, 5, 7, and 9 show the variation of entropy for different amount sampling points of wrist pulse signals. As shown in Figures 3, 5, 7, and 9, by increasing or decreasing the number of sample points, although there are fluctuations in the entropy values of some points, the overall fluctuation trend has not changed for different stages of pregnancy.

**Figure 4:**
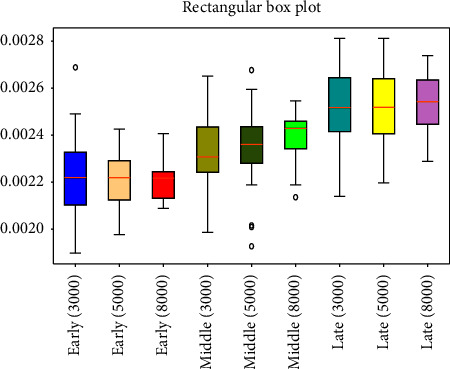
Box plots of FuEn in different stages of pregnancy.

**Figure 5:**
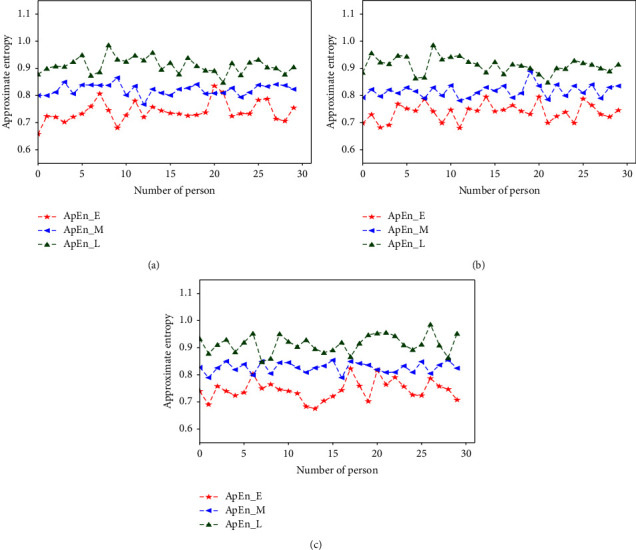
ApEn values of different pregnancy periods.

**Figure 6:**
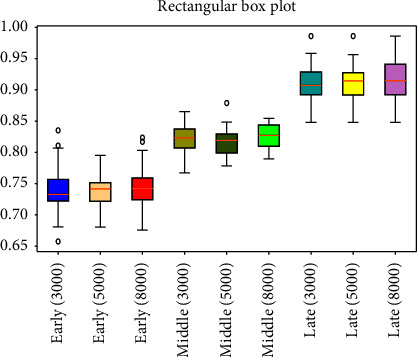
Box plots of ApEn in different stages of pregnancy.

**Figure 7:**
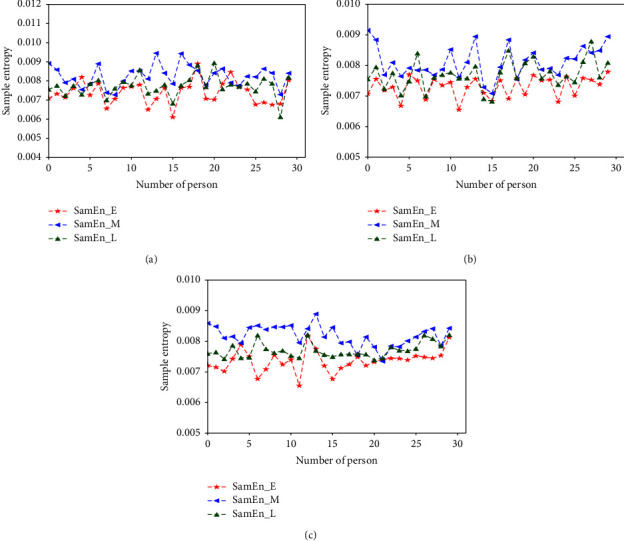
SamEn values of different pregnancy periods.

**Figure 8:**
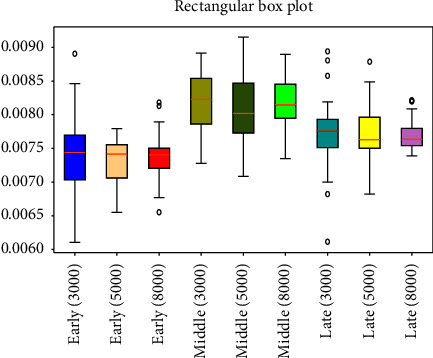
Box plots of SamEn in different stages of pregnancy.

**Figure 9:**
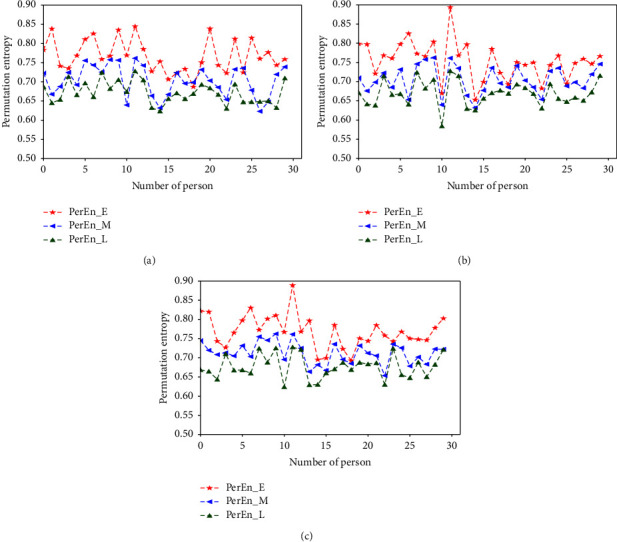
PerEn values of different pregnancy periods.

**Figure 10:**
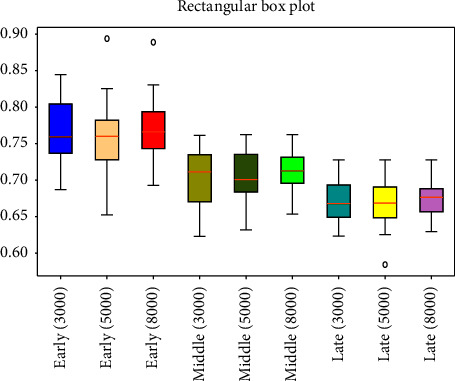
Box plots of PerEn in different stages of pregnancy.

This paper employed SPSS 25 statistical software to observe the entropy distribution of three groups' pulse signals. The variables between the groups were compared using the LSD-t test. A *p* < 0.05 was considered statistically significant.  Table 4 shows the entropy values and significance analysis for different pulse points. By observing Table 4, it is found that the *p* values of entropy were more than 0.05, which indicated that there was no significant difference between them. Also, the fluctuation trend of the four entropy among the three groups has not changed significantly. In addition, box plots are used to describe the discrete distribution of three groups' pulse signals, as shown in Figures 4, 6, 8, and 10. Median is chosen as an indicator in the statistical description, and 25% quartile and 75% quartile are used to describe the degree of dispersion.

### 4.3. Comparison of Different Acquisition Positions

In order to explain the change of entropy at different collection positions, the entropy values of Cun, Guan, and Chi positions of the left hand were calculated, respectively. As shown in Table 5, the entropy values of Cun, Guan, and Chi positions were given, ^∗^*p* < 0.05 versus E stage, ^∗^*p* < 0.05 versus M stage, ^•^*p* < 0.05 versus L stage, ^⋄^*p* < 0.05 versus three stages, respectively.

From Table 5, we can see that the ApEn of Cun has a significant difference between the E and L stages, which means that the degree of pulse disorder at the Cun position of the left hand in the L stage is greater than that in the E stage. Furthermore, the PerEn of the Cun of the left hand has a significant difference between the E and M stages and the E and L stages, respectively. It may be caused by major changes in the body of the pregnant woman as the pregnancy grows. For the Guan position of left hand, it can be clearly seen that only the PerEn has a significant difference between the E and M stages and the M and L stages, respectively. The FuEn and SamEn of the Cun and Guan positions showed slight fluctuations, which may be due to the fact that the pulse changes during pregnancy are not mainly reflected in these two positions. Compared with the Cun and Guan positions, the entropy values of the Chi position have obvious differences in each stage, which show that the pulse of the Chi position fluctuates greatly.

### 4.4. Time-Domain Features Analysis

**Figure 11:**
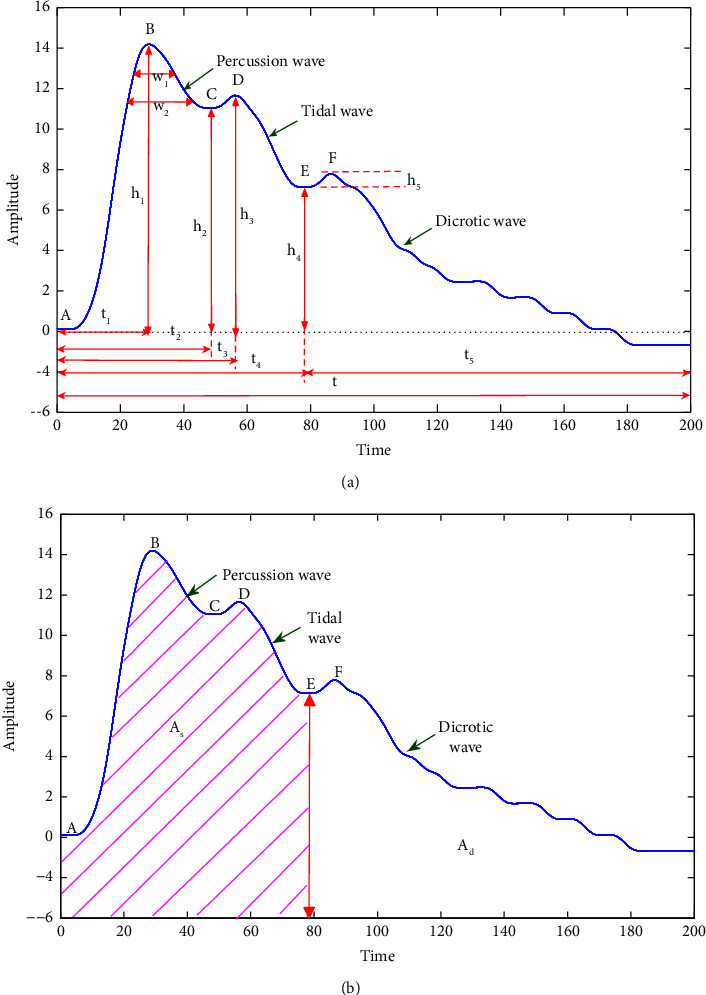
The time domain features of pulse.

Pulse features are generally calculated based on the peak width, as shown in Figure 11. Based on Li et al.‘s study [16], features are used to analysis for three stages of pregnancy.  Table 6 showed the time-domain parameters of Chi position of left hand during pregnancy. From Table 6, the results shown that *h2,h3* showed an increasing trend with pregnancy, while the *t2,t3,t,Ad* showed the opposite trend. Physiologically, it reflects the compliance of the aorta and the increased ejection capacity of the left ventricle. In addition, the significance analysis of the time-domain features of the three stages of pregnancy showed that the *p*_*EL*_-value of the t2, *p*_*EM*_-value and *p*_*EL*_-value of the *t* were 0.043, 0.003 and ≤0.000, indicating that there had greater changes between the *E* and L stage, *E* and M stage, respectively. Through the analysis of other time domain features, it is found that the features of each stage fluctuated in different degrees. However, the significant difference *p*-values were all greater than 0.05, which means that there were no major difference in each stage.

### 4.5. Comparison with Existing Pulse Signals Analysis

The entropy indicated that it quantifies some of the nonlinear features, such as multiplicity, rate of variability, time-irreversibility of time series. FuEn, ApEn, SamEn, and PerEn can track qualitative changes in time series patterns and allow one to assess the temporal regularity of the time series.

Compared with entropy analysis, the researches focus of existing works are the optimization of time-domain waveform parameters or frequency-domain spectrum parameters, few involved with complexity statistics. For example, Li et al. used time domain, frequency domain, time-frequency domain, high-dimensional feature analysis methods to study the correlation and difference of pulse characteristics of the wrist Cun, Guan and Chi position. The research found that pulse characteristics with high correlation at Cun-Guan-Chi position of left and right hands are mainly power spectrum characteristics and time-frequency characteristics, while the features with high correlation between both hands mostly concentrate in the time domain characteristics [16]. Zheng et al. quantify the difference of Gaussian modelling characteristics derived from radial pulses measured from the three trimesters of healthy pregnant women. They concluded that the second to third trimester, T1,2, decreased significantly and T1,3 and R1,3 decreased slightly but nonsignificantly [34]. Zhou et al. proposed a time series method to analyze health changes in participants. The correlation between pulse manifestation and health indicators was analyzed using a structural equation model [35]. Chu et al. determine a significant difference between pulse-taking depths and pulse-taking positions by peak value, power, ascending slope, and descending slope [36].

For entropy, when entropy analyzes the nonlinear coupling behavior of synchronized pulse time series, the complexity information of pulse and reproductive, cardiovascular, respiratory and other system can be obtained. For instance, Mischi et al. adjusted the estimates of ApEn and SamEn to monitor regular changes in single-channel EHG recordings reflecting electrohysterogram changes as pregnancy progressed [37]. Chen proposed and applied FuEn to the character of surface electromyography (EMG) signals. It shows that FuEn can more efficiently measure the regularity of time series [33]. Therefore, different from calculation methods such as time domain, frequency domain, trend, etc., entropy analysis can reveal some new and different results, which are worthy of further study.

### 4.6. Discussion

Pregnancy is a special physiological stage for women, which the internal body, blood circulation and internal organs of women will change in varying degrees during the period. TCM believes that the blood gathers to raise the fetus, the fetal Qi is agitated, the blood is vigorous, the blood volume increases, the blood is diluted, the cardiac output increases, the peripheral resistance is reduced, the blood velocity is accelerated, and the pulse condition becomes more slippery and powerful after pregnancy. This study demonstrated that the pulse of different periods of pregnancy will show varying degrees of change with gestational weeks, such as pulse rate, pulse speed, etc.

In general, FuEn, ApEn are all important indexes to evaluate the degree of confusion between the front and back parts of the pregnancy pulse. Through the analysis of the two kinds of entropy, it is clear that the degree of pulse disorder in L stage is greater than that in *E* and M stage. This study suggests that the third trimester is slightly characterized by a larger number of series manifesting nonlinear dynamics compared to the first and second trimester by using entropy as the discriminating statistic. Moreover, the two kinds of entropy show a high degree of consistency in the fluctuation trend. The SamEn showed a fluctuating trend of first increasing and then decreasing with the gestation period. One of the important reasons may be the systemic changes of blood pressure, peripheral resistance and pulse force of pregnant women due to infant development. However, the PerEn is opposite to the other three kinds of entropy. The PerEn, which is used to describe the mutation characteristics of system dynamics, shows that the human pulse changes greatly in the first trimester of pregnancy compared with the second and third trimester. The study shows that these parameters derived from radial pulse waveform had similar changes trend although there was some slight differences in pulse values.

From the entropy point of view, we can also judge some notable relationship of pulses variability with the gestational period. Importantly, we note that the entropy method has potential applications to study a wide variety of other physiologic and physical time series data. In summary, this study has quantified the pulse waveform characteristic differences in terms of pulse entropy among the three trimesters, providing useful scientific evidence to better understand the physical and psychological changes of body during pregnancy.

## 5. Future Work

The purpose of this paper is to use entropy to explore the changes of pulse during the pregnancy period. Compared the traditional methods with nonlinear analysis, more information and the inherent rules about pregnancy pulse can be found to verify the pulse changes of pregnant women by nonlinear methods.

Data collection may be affected due to differences in collection equipments and external environment interference. Given the small sample size of this study and the inherent difficulty of successfully recording pulse during pregnancy, our findings and interpretation should be confirmed and carefully considered. In the future, the more pregnancy pulse data will be collected in the three trimesters and try to use machine learning methods to establish the discrimination model of pulse in three pregnancy stages and the discrimination model of normal pregnant women and non-pregnant women. In addition, the traditional analysis methods such as time domain, frequency domain and time-frequency domain and different nonlinear methods will be used to analyze the data of gestation.

## Figures and Tables

**Table 1:** Mapping the Cun, Guan, and Chi displacements versus organs of the body.

Position	Cun	Guan	Chi
Left arterial wrist	Heart	Liver/Gallbladder	Kidney
Right arterial wrist	Lung/Chest	Stomach	Kidney

**Table 2:** Basic physiological data of the participants.

Characteristic	Number or Mean ± SD
Number(n)	90 (*E* = 30, *M* = 30, *L* = 30)
Age (year)	28.4 ± 3.5
Height (cm)	166.2 ± 7.3
Weight (kg)	64.5 ± 8.3
BMI (kg/*m*^2^)	20.6 ± 4.2
Systolic/diastolic blood pressure (mmHg)	115.5/67.8 ± 16.8/6.4

**Table 3:** The statistical analysis for different pregnancies.

	FuEn	ApEn	SamEn	PerEn
Mean ± Sd of *E* stage	0.00221±0.00013	0.73775±0.03218	0.00730±0.00034 0	0.75486±0.04991
Mean ± Sd of *E* stage	0.00235±0.00017	0.81687±0.02234	0.00811±0.00052	0.70502±0.03666
Mean ± Sd of *E* stage	0.00251±0.00015	0.91196±0.03013	0.00770±0.00046	0.66923±0.03306
*p* (*E* and M)	0.001	≤0.001	≤0.001	≤0.001
*p* (*E* and L)	≤0.001	≤0.001	0.001	≤0.001
*p* (M and L)	≤0.001	≤0.001	0.001	0.001

**Table 4:** The entropy values for different pulse points.

Pulse number	E stage				M stage				L stage			
	FuEn	ApEn	SamEn	PerEn	FuEn	ApEn	SamEn	PerEn	FuEn	ApEn	SamEn	PerEn
3000^1^	0.00227	0.74063	0.00739	0.76794	0.00233	0.82135	0.00818	0.70357	0.00251	0.91085	0.00771	0.67179
5000^2^	0.00221	0.73775	0.00730	0.75486	0.00235	0.81685	0.00811	0.70502	0.00252	0.91196	0.00770	0.66923
8000^3^	0.00221	0.74386	0.00736	0.76736	0.00241	0.82694	0.00819	0.71245	0.00254	0.91432	0.00771	0.67824
*p*_12_	0.698	0.755	0.474	0.267	0.690	0.404	0.563	0.876	0.998	0.890	0.820	0.749
*p*_13_	0.683	0.726	0.842	0.961	0.057	0.298	0.967	0.337	0.535	0.667	0.857	0.420
*p*_23_	0.984	0.508	0.605	0.288	0.130	0.063	0.536	0.421	0.533	0.770	0.963	0.261

**Table 5:** The entropy values for different acquisition positions.

Position	E stage				M stage				L stage			
	FuEn	ApEn	SamEn	PerEn	FuEn	ApEn	SamEn	PerEn	FuEn	ApEn	SamEn	PerEn
Cun	0.00211	0.81809	0.00721	0.71115^∗•^	0.00202	0.82765	0.00720	0.65970^∗•^	0.00200	0.83554^∗^	0.00716	0.67473^∗^
Guan	0.00206	0.82444	0.00727	0.70735^∗^	0.00203	0.83197	0.00735	0.63832^∗^	0.00202	0.82943	0.00717	0.68018^∗^
Chi	0.00221^⋄^	0.73775^⋄^	0.00730^⋄^	0.75486^⋄^	0.00235^⋄^	0.81687^⋄^	0.00811^⋄^	0.70502^⋄^	0.00251^⋄^	0.91196^⋄^	0.00770^⋄^	0.66923^⋄^

**Table 6:** The pulse times domain features of the Chi position of the left hand.

Pulse number	E stage	M stage	L stage,	*p*_*EM*_	*p*_*EL*_	*p*_*ML*_
h1	13.829	14.925	14.717	0.275	0.375	0.831
h2	10.891	11.515	11.821	0.437	0.248	0.703
h3	10.456	10.933	11.322	0.554	0.284	0.629
h4	6.000	6.133	5.812	0.796	0.715	0.533
h5	0.051	0.048	0.117	0.640	0.425	0.741
t1	0.114	0.113	0.116	0.720	0.478	0.725
t2	0.183	0.176	0.172	0.233	0.043	0.395
t3	0.200	0.197	0.190	0.544	0.065	0.210
t	0.725	0.652	0.630	0.003	≤0.001	0.349
W1	0.136	0.125	0.139	0.851	0.948	0.800
W2	0.084	0.125	0.089	0.324	0.881	0.402
As	71.132	72.337	69.775	0.825	0.804	0.639
Ad	32.457	30.541	27.788	0.555	0.153	0.379

## Data Availability

The data used to support the findings of this study are included within the article. The experimental data were collected in the Affiliated Obstetrics and Gynecology Hospital of Fudan University in this paper.
